# 

**Published:** 1896-10

**Authors:** 


### CONTENTS.

ORIGINAL COMMUNICATIONS—
THE NON-OPERATIVE TREATMENT OF URETHRAL STRICTURES. By Fred C. Valentine, M.D., New York ..... 505
SANITARY REGULATIONS AND PREVENTION OF TUBERCULOSIS. By Katharine R Collins, M.D., Atlanta, Ga ..... 511
SUPPLEMENTARY COURSES OF TREATMENT REQUIRED FOR CHRONIC NASAL INFLAMMATION. By Thos. F. Rumbold, M.D., St. Louis, Mo ..... 516
BROKEN BONE-SHAFTS. By Thos. H. Manley, M.D., New York ..... 521
ELECTRICITY IN GYNECOLOGY AT THE HOWARD HOSPITAL, PHILADELPHIA. By G. Betton Massey, M.D., Philadelphia ..... 524
NITRIC ACID AND GUM CAMPHOR IN THE TREATMENT OF CHANCROID. By Lucien Lofton, M.D., Atlanta, Ga ..... 528
SOME OF THE UNSUSPECTED EFFECTS OF THE BROMIDES. By J. Allison Hodges, M.D., Richmond, Va ..... 529

SOCIETY REPORTS—
RICHMOND ACADEMY OF MEDICINE AND SURGERY ..... 537
SECTION ON OPHTHALMOLOGY ..... 547

CORRESPONDENCE—
MEDICAL NEW YORK ..... 553

EDITORIAL—
DRINKING-WATER ..... 557
A MEDICAL OFFICER OF HEALTH FOR GEORGIA ..... 559
OCULIST OR OPTICIAN ..... 562
THE SUBSCRIPTION PRICE OF MEDICAL JOURNALS AND THE SAMPLE COPY EVIL ..... 563

SELECTIONS AND ABSTRACTS—
SKIN DISEASES AND SYPHILIS ..... 565
TOO MANY COLLEGES ..... 568
PEROXIDE OF HYDROGEN ..... 569

BOOK REVIEWS ..... 572
MEDICAL ITEMS—CONTINUED ..... x, xi, xii, xiii
ADVERTISERS' INDEX TO ADVERTISEMENTS ..... xiv
CLASSIFIED INDEX TO ADVERTISEMENTS ..... xxxiii

